# User-Centered Design of an Electronic Dashboard for Monitoring Facility-Level Basic Emergency Obstetric Care Readiness in Amhara, Ethiopia: Mixed Methods Study

**DOI:** 10.2196/64131

**Published:** 2025-04-03

**Authors:** Kylie Dougherty, Yihenew Tesfaye, Heran Biza, Mulusew Belew, Natalie Benda, Abebe Gebremariam Gobezayehu, John Cranmer, Suzanne Bakken

**Affiliations:** 1 Department of Medical Social Sciences Feinberg School of Medicine Northwestern University Chicago, IL United States; 2 College of Health Sciences Bahir Dar University Bahir Dar Ethiopia; 3 Nell Hodgson Woodruff School of Nursing Emory University Atlanta, GA United States; 4 Emory-Ethiopia Partnership Bahir Dar Ethiopia; 5 School of Nursing Columbia University New York, NY United States; 6 Woodruff Health Sciences Center Emory University Atlanta, GA United States; 7 Center for the Study of Human Health Emory University Atlanta, GA United States

**Keywords:** health information technology, design and evaluation, Ethiopia, usability, nursing informatics, user-centered design, basic emergency obstetric care, obstetric, nurse, user-centered, design, maternal mortality, maternal, develop, sub-Saharan Africa, Africa, dashboard, tracking, emergency care

## Abstract

**Background:**

Maternal mortality remains a persistent public health concern in sub-Saharan African countries such as Ethiopia. Health information technology solutions are a flexible and low-cost method for improving health outcomes with proven benefits in low- to middle-income countries’ health systems.

**Objective:**

This study aimed to develop and assess the usability of an electronic dashboard to monitor facility-level readiness to manage basic emergency obstetric care (BEmOC) in Amhara, Ethiopia.

**Methods:**

The study used three methods to iteratively refine the dashboard: (1) user-centered design sessions with individuals who interact with the BEmOC supply chain, (2) review and feedback from domain and information visualization subject matter experts (SMEs) to refine the dashboard, and (3) usability heuristic evaluation with human-computer interaction (HCI) SMEs.

**Results:**

User-centered design sessions resulted in a preliminary version of the dashboard informed by end-user preferences and perceptions, with recommendations focusing on aesthetic design, filtering and sorting, and matching with the real world. An example of an end-user recommendation included increasing font sizes on the dashboard and using a red, yellow, and green color-coding scheme. Next, domain and visualization SMEs continued the dashboard’s iterative refinement, focusing on aesthetic design and navigation, by confirming design choices incorporated from the user-centered design sessions and recommending changes to enhance user experience moving through the dashboard, such as adding more filtering options. HCI SMEs rated the dashboard as highly usable (0.82 on a scale of 0-4, with 0 being no usability concern and 4 being a catastrophic usability concern). The principle with the highest usability severity scores was a match between the system and the real world with a score of 1.4. The HCI SMEs also rated the information visualization aspects of the dashboard favorably with 2 usability principles, spatial organization and information coding, scoring 0.

**Conclusions:**

Dashboards are a novel method for promoting and tracking facility capacity to manage BEmOC. By including targeted end users and SMEs in the design process, the team was able to tailor the dashboard to meet user needs, fit it into the existing government health systems, and ensure that the dashboard follows design best practices. Collectively, the novel, customized BEmOC dashboard can be used to track and improve facility-level readiness in Amhara, Ethiopia, and similar global BEmOC facilities.

## Introduction

Maternal mortality is a critical public health issue, particularly in low-resource settings like sub-Saharan Africa, which accounts for over three-fourths of global maternal deaths [1]. Despite progress in reducing adverse maternal outcomes in countries like Ethiopia, high maternal mortality ratios persist, largely due to gaps and stockouts of essential supplies for managing obstetric emergencies [2,3]. Inadequate supplies for basic emergency obstetric care (BEmOC) can lead to delayed or suboptimal care. A study on facility-level readiness to manage BEmOC in Amhara, Ethiopia, using the Clinical Cascades reported a 63.3% mean overall BEmOC readiness for managing the 6 most common emergencies, which underscores the need for improving facility-level BEmOC readiness in this region [4].

A promising approach to improve facility-level BEmOC readiness is the development and implementation of health information technology (HIT) to monitor critical supply availability. HIT involves the processing, storage, and exchange of health information in an electronic environment, and has been shown to improve health outcomes and strengthen health systems in low-resource settings [5,6]. Electronic dashboards are a form of HIT that can be used to track critical information, provide alerts, assess performance indicators, develop reports, and customize data views, which makes them a useful tool for monitoring inventory data and facility-level readiness [7,8]. For example, dashboards were used in Ethiopia to monitor the supply inventory of malaria medications and prevent stockouts from occurring, and a simple dashboard was merged into the existing Integrated Pharmaceutical Logistics System (IPLS) in the Amhara and Oromia regions of Ethiopia, resulting in improved facility-level readiness to treat tuberculosis by ensuring health care facilities had adequate supplies [7,8]. Beyond Ethiopia, HIT has been used to anticipate medical stockouts and improve maternal health outcomes in low-resource settings [9,10]. This evidence highlights the potential for HIT to improve facility-level BEmOC readiness in Amhara, ultimately contributing to improved maternal care quality and reduced maternal mortality ratios.

Previous studies, including those conducted in low-resource settings and Ethiopia, have demonstrated the benefits of HIT and dashboards in improving facility-level readiness [5-8]. Furthermore, the growing utilization of technology and cellular services across Africa further supports the feasibility of implementing successful electronic solutions, driven by the increasing adoption of HIT [11,12]. Finally, Ethiopia has recognized the value of HIT in strengthening health care systems and has been implementing electronic medical records in its health care facilities since 2013 [13].

However, while dashboards can be a powerful tool for improving health outcomes, their success depends on ensuring high usability, enabling end users to effectively accomplish their tasks. Usability is defined as “the extent to which a product can be used by specified users to achieve specified goals with effectiveness, efficiency, and satisfaction in a specified context of use” [14]. Despite the potential of HIT, many implementations have failed due to insufficient consideration of end users’ needs and the environment in which the technology is applied [15,16]. Similarly, while dashboards show promise for improving facility-level BEmOC readiness, their success relies on development that incorporates input from targeted end users, accounts for the context of use, and adheres to usability principles. This study aims to develop and assess the usability of an electronic dashboard for monitoring facility-level BEmOC readiness.

## Methods

### Overview

To create the initial version of the dashboard, the team incorporated findings from previous qualitative interviews with individuals who worked in Ethiopia’s obstetric emergency supply chain, used information and designs from the preexisting dashboards in Ethiopia’s IPLS, and followed best practices found in the current literature for dashboard development [17-20]. The initial dashboard and its components were developed in Microsoft PowerPoint (refer to Multimedia Appendix 1).

To ensure adequate fit between the dashboard, the required tasks, the current system, the environment, and intended users, the research team conducted participatory user-centered design sessions, performed informal evaluations with domain and information visualization subject matter experts (SMEs), and completed a heuristic usability evaluation with human-computer interaction (HCI) SMEs.

### Ethics Considerations

The research team obtained ethics approval from Columbia University’s institutional review board (IRB) (IRB-AAAU2006), Emory University’s IRB (MOD005-STUDY00005335), and the Amhara Public Health Institute (NoH/RfftTlDlo7l44). Before collecting data, the research team provided participants with an information sheet and gave them time to ask questions about the study. Once all questions had been answered, the team obtained verbal informed consent from all participants before collecting data. All ethics review boards waived the requirement for written documentation of informed consent because the study procedures met the criteria for minimal risk to participants. All data was de-identified and participants data was linked to a random identification number. Only KD had access to the list of participant names linked to the ID numbers. None of the targeted end-users, domain experts, or subject matter experts received compensation for their participation in the study. Human-computer interaction experts participating in the heuristic evaluation received a $50 amazon gift card for their participation.

### Sample Selection

#### User-Centered Participatory Design Sample Selection

Consistent with existing literature in the field of participatory design, the team estimated that a sample size of 15 would be sufficient to reach design saturation, a state where the design sessions reveal no major revisions to the designs reviewed [21,22]. The research team purposively recruited individuals with firsthand experience working within the BEmOC supply chain in Amhara, Ethiopia, at both the regional and local or facility levels. Participants were required to be at least 18 years old. Study participants were Ethiopian citizens and full-time employees in one aspect of Amhara’s BEmOC supply chain, such as hospital supply managers, regional hub employees, and pharmacists.

#### Informal Expert Review Sample Selection

The domain SME was a research team member with over 6 years of experience conducting research in Amhara, Ethiopia; evaluating the supply chain; and measuring facility-level readiness to manage BEmOC. The information visualization SMEs were individuals from Columbia University School of Nursing’s Visualization Design Studio, where individuals with varying levels of expertise can learn and gain advice on their current projects. The discussions at this studio were led by 2 PhD-level faculty, a nurse scientist with expertise in visualization design and evaluation, and a human-factors engineer. Key contributions also came from other faculty members at the university who have training in the areas of nursing research and biomedical informatics. All information visualization SMEs have published in the areas of information visualization, HCI, and user experience.

#### Heuristic Evaluation Sample Selection

The study sample included 5 HCI SMEs who were recruited through the team’s professional networks. Previous research has found that 5-8 SMEs can identify over 80% of HIT usability violations [23,24]. HCI SMEs were eligible for inclusion if they had conducted research or published in the field of user interfaces or information visualizations and had not previously evaluated the dashboard.

### Procedures

#### User-Centered Design Procedures

Staff from Emory-Ethiopia Partnership and Amhara Regional Health Bureau identified BEmOC supply chain stakeholders and reached out to determine if they were interested in participating in this study. A member of the research team contacted interested individuals via phone call, email, and WhatsApp (Meta) messaging to explain the study in detail, answer any questions, and set a time to meet in person with the participant to obtain verbal consent and perform the research activities. We used multiple communication channels to reach potential participants; in particular, WhatsApp has been found to be a useful tool for recruiting participants, specifically in global settings [25,26]. Design sessions were conducted in English and Amharic, and data collection tools were available in both languages since Amharic is the national language and Ethiopia’s health care professionals receive clinical training in English [27,28].

Design sessions lasted approximately 60 minutes and occurred in small groups of 2-4 people within the health care facility or health bureau offices. Participants were given printed copies of the initial dashboard and asked to verbalize their thought processes as they explored the visualizations and information in the preliminary dashboard [29-32]. Design sessions were audio recorded and transcribed into English for analysis by a team member who is bilingual in Amharic and English. The individual conducting the analysis is a native Amharic speaker with over a decade of experience translating Amharic to English for health care research. Given their extensive expertise and the time constraints of the study, the team opted not to perform back translations. The dashboard development followed an iterative process, consisting of 4 design sessions. After each session, the research team incorporated recommended changes into the dashboard, which were then presented in the subsequent sessions [30].

#### Informal Expert Review Procedures

The team presented the Microsoft PowerPoint dashboard to a domain expert and a group of information visualization SMEs. Team member (KD) used Zoom (Zoom Video Communications) screen-sharing capabilities to walk the domain expert through the dashboard and their various features. The domain expert then reviewed the dashboard and provided recommendations for improvement.

KD presented the same dashboard to information visualization SMEs at 2 sessions of the Visualization Design Studio [33]. KD walked the SMEs through the dashboard, answered questions about their intended use, and elicited feedback and recommendations. Collectively, both rounds of feedback were used to optimize and further develop a medium-fidelity version of the dashboard for use in the heuristic usability evaluation.

#### Heuristic Evaluation Procedures

The team used Figma to develop a medium-fidelity prototype, which allowed the simulation of the dashboard’s workflow [34]. Using Zoom Pro, a Health and Insurance Portability Accountability Act (HIPAA)–compliant videoconference platform, and its screen-sharing technology, KD asked the HCI SMEs to complete several tasks using the dashboard and encouraged them to explore other components of the dashboard (Multimedia Appendix 2). During their exploration, participants completed a heuristic evaluation checklist to identify and rank the severity of the usability violations and had the opportunity to explain the violations they encountered [23,24,32]. They were also asked to describe what they were thinking, feeling, and seeing while completing the various tasks. The sessions were video recorded, and KD took field notes. At the end of the sessions, the HCI SMEs and KD discussed potential solutions for the identified usability violations. Participants were asked to complete tasks during the heuristic usability evaluation mentioned in Textbox 1.

**List of tasks participants were asked to complete during the heuristic usability evaluation.**

**Heuristic usability evaluation tasks**

- Filter the main screen to only view primary hospitals.
- Determine which health care facilities are not ready to manage retained placenta.
- Determine if [hospital name] is ready to manage hypertensive emergencies. If not, identify which items are missing.
- Determine if [hospital name] is ready to manage maternal sepsis or infection. If not, identify which items are missing.
- Determine what emergencies [hospital name] is ready to manage.
- For prolonged labor at [hospital name] filter the supplies table to see the items that are at the emergency order point.
- For prolonged labor at [hospital name] filter the supplies to see the items that are overstocked.
- Determine how many health care facilities are at risk for not being able to manage retained placentas.

The Heuristic Evaluation Checklist used in this study was guided by Nielsen’s 10 usability heuristics (ie, best practices; Table 1) [24,35] and includes a 5-item scale where experts rate each usability heuristic on a scale of 0 (not a problem) to 4 (catastrophic violation; Table 2) [23,32,36]. The research team modified the original checklist to fit the needs of a medium-fidelity dashboard (Multimedia Appendix 2). For example, questions for heuristics related to error prevention, help users with errors, and help and documentation were excluded since those tasks could not be simulated with the medium-fidelity prototype. In addition, 3 usability heuristics specific to information visualizations in the dashboards were included: spatial organization, information coding, and orientation [19].

**Table 1:** Usability heuristics and their definitions used in the heuristic usability evaluation.

Usability heuristic	Type	Definition
Visibility of system status	HIT^a^	The system should always keep users informed about what is going on through appropriate feedback in a reasonable time.
System and real-world match	HIT	The system should speak the user’s language, with words, phrases, and concepts familiar to the user. The system should follow real-world conventions and ensure the dashboard fits within the existing workflow and technology system.
User control and freedom	HIT	Users should be free to select and sequence tasks and make their own decisions regarding the cost of exiting current work. Users should have clearly marked “emergency exit” to leave the unwanted state.
Consistency and standards	HIT	Users should not have to wonder whether different words, situations, or actions mean the same thing. Systems should maintain interface design choices in similar contexts and differ in different contexts.
Recognition rather than recall	HIT	The user should not have to remember information from one part of the dialogue to another. Objects, actions, and options should be easily visible, and instructions should be visible or easily retrievable whenever appropriate.
Flexibility and efficiency of use	HIT	The system should offer users several options for finding content. Users should be able to customize their interface and achieve their goals in an efficient manner and have the capacity to adapt to users’ needs.
Aesthetic and minimalist design	HIT	The main dashboard should not contain information that is irrelevant or rarely needed. The system should present the largest amount of data with the least amount of ink.
Spatial organization	Information visualization	The overall layout of a visual representation should make it easy for the user to locate an information element in the display.
Information coding	Information visualization	The symbols and numbers used in the visualization should aid perception. The numeracy and graph literacy of the visualization should match the intended users’ ability.
Orientation and help	Information visualization	The system should provide support for the user and help to orient them in their visualization.

^a^HIT: health information technology.

**Table 2:** Severity ratings for usability violations in the heuristic evaluation.

Score	Usability problem	Explanation
0	Not a usability problem at all	No problem identified
1	Cosmetic problem	Need not be fixed unless extra time is available on the project
2	Minor problem	Fixing this should be a low priority
3	Major usability	Important to fix, so should be given high priority
4	Usability catastrophe	Imperative to fix this before the product can be released

### Data Analysis

#### User-Centered Design Analysis

Analysis was concurrent with data collection with modifications of the dashboard occurring after the initial 2 design sessions and following the third and fourth sessions. Using the translated transcripts of audio-recorded sessions and field notes, KD extracted key preferences, identified poorly performing graphics and dashboard components, and improved the design based on participant feedback. The team maintained detailed notes on design decisions and the rationale for those decisions alongside the notes and transcripts, which functioned as an audit trail.

#### Heuristic Usability Evaluation Analysis

The responses on the Heuristic Evaluation Checklist were transferred into Microsoft Excel for analysis [19,20]. The research team used unique participant numbers to link the responses to a confidential participant list. The data were analyzed with descriptive statistics (frequencies and means) [37]. Both session field notes and qualitative responses reported in the free response comments section of the checklist were used to understand usability and enhance the dashboard’s design. During the evaluations, KD documented key areas of concern that HCI SMEs verbalized during the sessions. Combing those notes with the free-response comments, KD used the frequency that those concerns were mentioned to understand their importance. The usability concerns and potential solutions reported from the free-response comments were also compiled into a single list of action items, with duplicates removed, and KD used this list to guide the refinement of the next version of the dashboard.

## Results

### User-Centered Design Sessions

#### Sample Characteristics

In total, 6 individuals, who work at the regional health bureau or regional hubs for supply distribution, participated in the first 2 design sessions and reviewed the initial version of the dashboard. Following the refinement of the dashboard by KD, 5 individuals who work at health care facilities in the region participated in the third and fourth design sessions and reviewed the updated version of the dashboard. Recruitment and data collection ended prematurely at 11 participants due to civil unrest and safety concerns in the region [38].

#### Evolution of the Dashboard Design

During design sessions 1 and 2, participants reviewed the initial version of the prototype, and during design sessions 3 and 4, they reviewed a revised version that incorporated feedback from study participants in design sessions 1 and 2. During design sessions 1 and 2, participants viewed the initial design of the dashboard and were able to easily grasp the idea the dashboard was trying to portray related to facility readiness to manage BEmOC. The users felt the dashboard, specifically, the regional view, helped them to understand which facilities would be ready to handle the emergencies. One regional respondent stated,

This image [dashboard] demonstrates how they [individual health care facilities] would be able to handle each health issue considering the level of the supplies that each hospital has for these health problems. In general, we can conclude from this image [regional dashboard] that some hospitals are in good condition for these health issues and others are not in good condition for these health issues.

Generally, participants in the first two sessions focused on (1) individual graphical component preferences such as which type of charts they preferred to see the data presented and (2) how they would want the data to be filtered and sorted. The third and fourth sessions with the refined dashboard focused on (1) confirming the terminology in the dashboard, and that it matched terms used in the supply chain, and (2) affirming design changes that were incorporated from the earlier sessions. Respondents in the third and fourth sessions also provided feedback on which job roles should have access to the dashboard. A participant during the fourth design session stated,

This dashboard [emergency-specific view] is essential for the dispensary unit as well. The store and supply officer bears most of the workload or duty in this workplace, but if the pharmacists and hospital managers have access to the data, it would be very good.

The changes that were incorporated during the design sessions, as well as the session number, iterations of the dashboard, and job level that provided the recommendations, can be found in Multimedia Appendix 3. In the following paragraphs, more details are provided on aesthetic design, filtering and sorting, and matching with real-world preferences.

#### Aesthetic Design

For colors used throughout the dashboard, participants wanted to use multiple colors that were consistent with those currently used in the existing dashboard. Furthermore, participants in the first 2 design sessions identified pieces of information that they thought should be removed from the dashboard because they did not assist the end users with making decisions related to supply ordering or maintaining facility-level readiness. However, to ensure users could find information that was not included in the dashboard, the research team added a button that would allow users to view the comprehensive drug list, which is already embedded in the electronic components of the existing IPLS. Facility-level participants, during design sessions 3 and 4, explained that not all health posts will manage certain obstetric emergencies, such as sepsis, so they should not receive readiness classifications for those emergencies. In response to this, the research team grayed out the classifications for those facilities and their corresponding emergencies.

Another aesthetic preference in the early design sessions included requests to include keys in the dashboard that define the colors and terms*.* Participants in design sessions 3 and 4 agreed that the keys assisted in their comprehension of the dashboard. Participants in design sessions 3 and 4 affirmed the design choices of the previous sessions and provided only small recommendations for the overall look of the dashboard, such as increasing the font size. Related to aesthetic decisions, design saturation was met with the smaller sample size.

#### Filtering and Sorting

Participants in the early sessions wanted to ensure there were multiple ways to filter the data so they could view pieces of information that were most important to them. Preferred filtering and sorting options included viewing inventory alphabetically within preestablished categories from the Ethiopian Pharmaceutical Supply System (eg, medical equipment, pharmaceuticals, and supplies), filtering supplies based on quantity available at the facility, and filtering facility by health care facility tier (eg, primary, general, referral, and center) [17]. Participants during the third and fourth design sessions endorsed the filtering options identified in the earlier sessions.

The participants from design sessions 3 and 4 also wanted employees at individual facilities to be able to view inventory data at other facilities besides their own. They felt having access to these data could support transferring excess supplies from one facility to another when neighboring facilities faced stockouts or were at an emergency order point. Thus, the research team added the ability for facility-level users to select different facility readiness data, similar to the regional-level users. Since there was very little new information identified during the second 2 design sessions, the research team determined that the study achieved design saturation for filtering preferences even with the smaller respondent sample.

#### Match With the Real-World Preferences

A feature that was a very important point of discussion in all the design sessions was using appropriate terminology that aligned with the participants’ current workflow and job expectations. Participants wanted to ensure that all the terms used within the dashboard aligned with terms from the IPLS. For example, supply names and categories as well as terms for quantities available were updated (eg, overstock changed to excess, and normal changed to functional for the category medical supplies).

One important change to note is that during the third design session, participants recommended changing the terminology for BEmOC readiness from ready, at risk, and not ready to normal, emergency order, and stockout so they would be the same as the terms used with the emergency-specific dashboard view. Based on this recommendation, the research team created 3 different readiness terminology keys. The 3 options included the original terms; the newly recommended terms; and a third option of yes, no, and emergency order. Since the team could not confirm their preferred choice for readiness terminology due to the civil conflict in the region, the domain expert selected the final terms—yes, at risk, and no. Since the team was unable to obtain user preferences related to this final change, the study did not achieve design saturation in the “match with the real-world” domain.

#### Final Design

**Figure 1:**
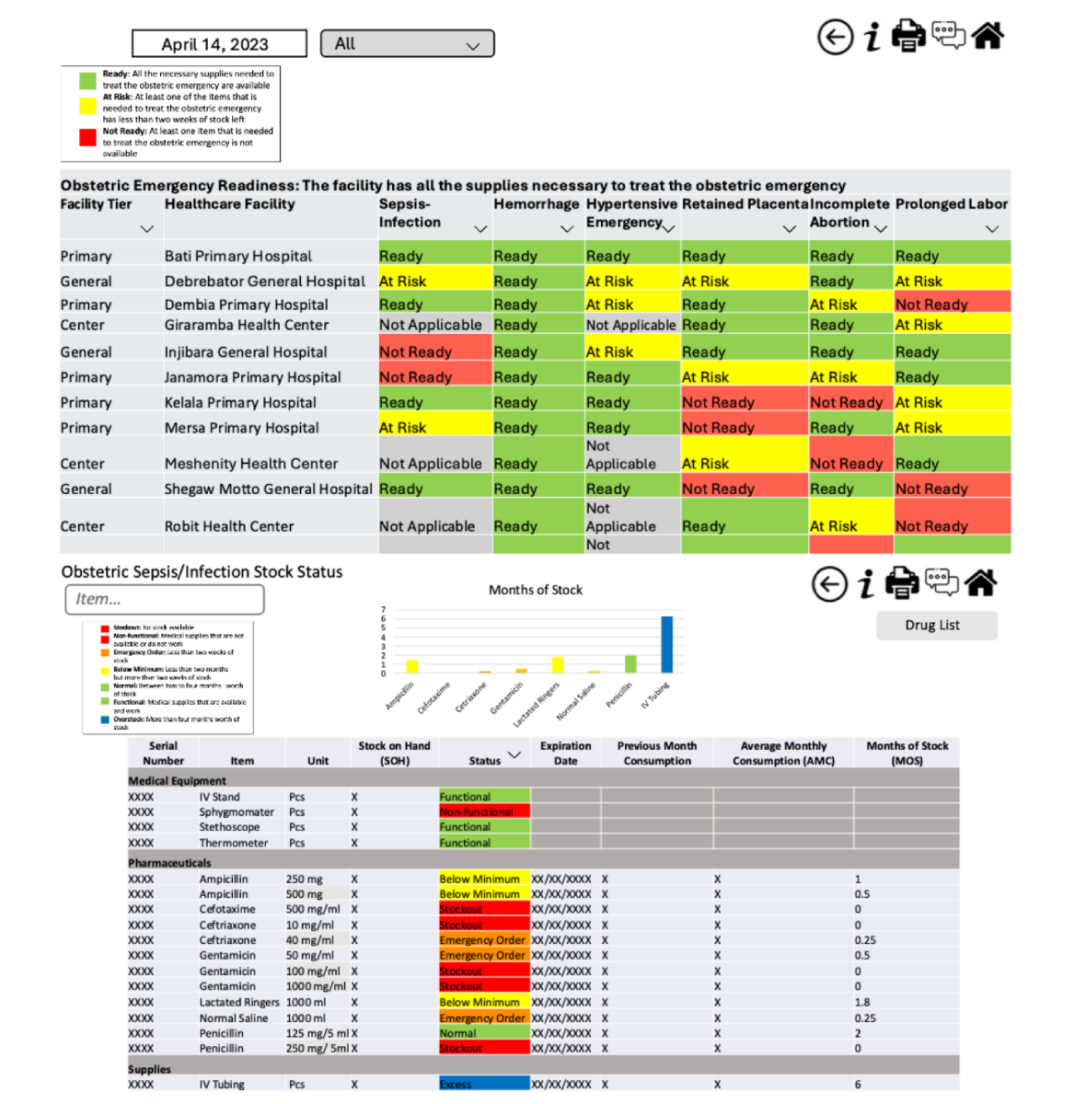
Regional basic emergency obstetric care (BEmOC) readiness dashboard and emergency-specific, facility-level dashboard following user-centered design sessions. Hospital names are real, but all readiness data are fictitious.

The final design comprised 2 different dashboard views for monitoring BEmOC readiness—one for the regional level and another for the facility level. The regional-level dashboard view provides a summary of readiness for the 6 obstetric emergencies for all health care facilities in the region. The facility-level dashboard view focuses on one facility and one obstetric emergency at a time. Overall study participants viewed the dashboard positively, with one respondent from design session 4 reporting “The hard copies [of the dashboard] you showed us today are beautiful and wonderful.” Figure 1 shows the design of the dashboard following the integration of the findings from the third and fourth user-centered design sessions, and Multimedia Appendix 3 lists all the changes that were incorporated into the dashboard following the user-centered design sessions.

### Informal Expert Review

The domain expert’s largest contribution to the refinement of the dashboard was his input on which terms to use to define BEmOC readiness, specifically for the regional view dashboard. To make the final decision on readiness classification terms that were discussed in the final user-centered design session, KD presented the domain expert with 3 different options created following the fourth design session. After reviewing the options, the domain expert recommended KD use yes, emergency order, and no. The rationale for this decision was the yes and no choice was the simplest option, and he believed the end users would be able to readily act based on these classification terms. The domain expert also affirmed KD’s decisions to group data at the regional and facility levels and felt that no critical information was missing from the dashboard.

**Figure 2:**
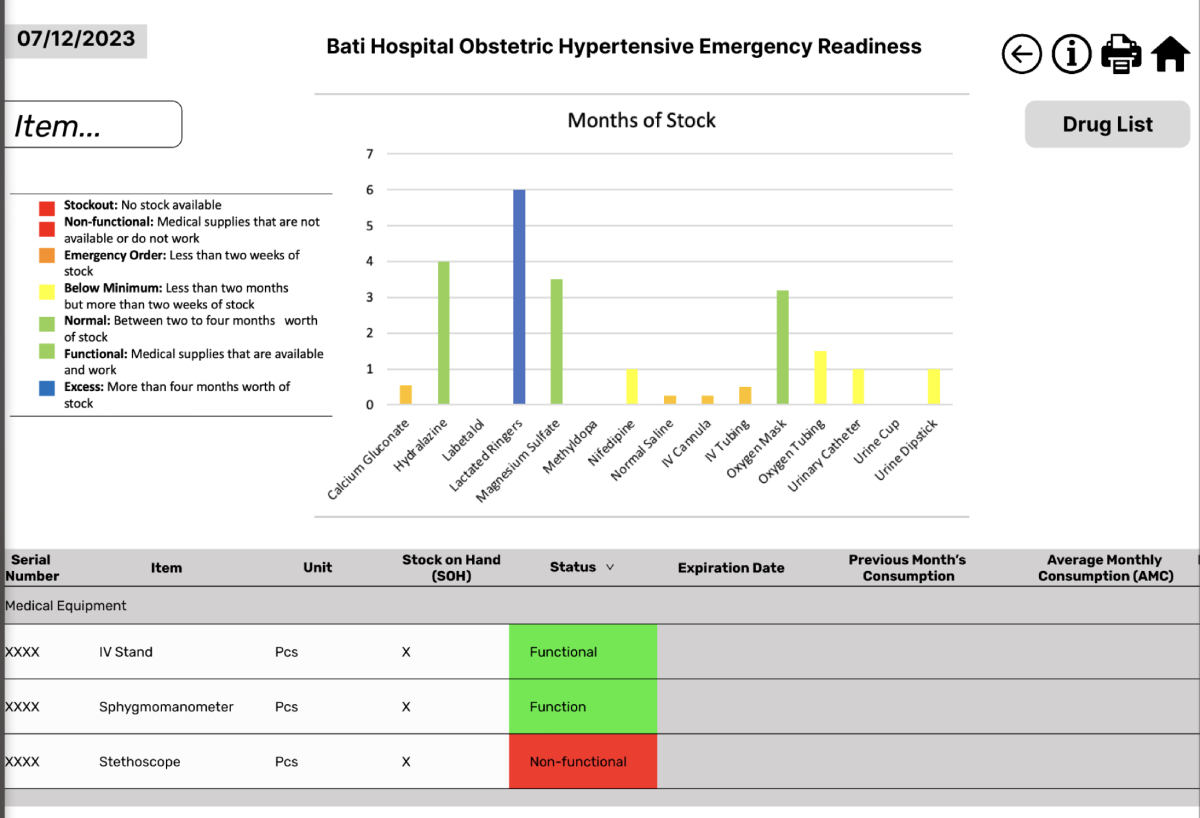
Regional view dashboard following informal expert review. All inventory data in this dashboard are fictitious.

During the review by information visualization SMEs, most recommendations aesthetics related to aesthetics and navigation. In terms of aesthetic design, the recommendations mainly focused on where to place different pieces of information or graphics to best grab the users’ attention or prevent overcrowding on a screen, for example, moving several columns of data to the right in the emergency-specific dashboard view. This would allow users to scroll horizontally to see that information, but it would not be visible without scrolling so there would be less information crowding the page. To improve navigation between the various dashboard views, the SMEs during the design studio recommended adding additional filtering options, such as for emergency readiness categories. They also recommended adding an affordance feature to the dashboard so that users would be able to see which items on the screen are clickable or not. In addition, the experts recommended adding several additional titles to different dashboard views to enhance clarity. These changes were incorporated when the dashboard was updated from PowerPoint to Figma (Figure 2).

### Heuristic Usability Evaluation With Human-Computer Interaction Experts

The heuristic evaluation took approximately 30 minutes per session. The years of experience for HCI SMEs ranged from 4 to 14 years, with a mean of 9. All HCI SMEs were female.

Overall, severity scores for heuristic evaluation ranged from 0 to 3 for the 10 usability principles. The mean usability score for all ten heuristics combined was 0.82 (SD 0.98), with a mode of zero. The principle with the highest severity rating was a match between the system and the real world, with a score of 1.4, which falls between a minor and cosmetic usability concern. For this principle, 4 HCI SMEs identified inappropriate color choices for both potential users with color blindness and using colors not congruent with common color-coding expectations as a common issue. Additional problems included the dashboard using concepts and phrases that were unfamiliar to the evaluators, and 3 evaluators felt the section headings were not ordered in the most logical fashion. HCI SMEs identified issues with usability principles visibility of system status, user control and freedom, consistency and standards, and recognition rather than recall (mean 1.2 for each, SD 0.84, 1.3, 0.84, and 1.3, respectively), which also falls between a cosmetic and minor usability concern. Concerns related to these principles included 2 of the HCI SMEs identifying the lack of clear exits for every dashboard and feeling that the current design hindered their ability to efficiently navigate the system. Table 3 shows the mean value, range, and mode for the 10 heuristic principles.

**Table 3:** Identified heuristic usability problems and their severity scores.

Heuristic category	Problems	Score, mean (SD)	Score, range	Score, mode
Visibility of system status	Title font is too small Color choices may be inappropriate for color-blind users Vertical bar charts make reading supply labels more difficult Need hyperlinks to improve navigation Menu-naming terminology is not consistent with the user’s task domain	1.2 (0.84)	2	1 and 2
Match between system and real-world	Color choices may be inappropriate for color-blind users Section headings and subheadings are not ordered in the most logical way Not all words, concepts, and phrases were familiar to the human-computer interaction subject matter experts Some of the colors used in the dashboard do not correspond to common expectations about color codes	1.4 (0.89)	2	1
User control and freedom	Need hyperlinks to improve navigation Not a clear exit on each dashboard screen Not all screens are accessible across the system Users could not easily move forward and backward between fields	1.2 (1.3)	3	0
Consistency and standards	Titles on the regional view dashboard do not update when the user filters the data Abbreviations not clearly explained Some colors are too similar to distinguish from other categories Not enough or inconsistent visual cues to identify active screens	1.2 (0.84)	2	1 and 2
Recognition rather than recall	White space is not optimized within the emergency-specific dashboard view Prompts, cues, and messages are not placed where the eye is likely to be looking on the screen	1.2 (1.3)	3	0
Flexibility and efficiency of use	Need hyperlinks to improve navigation	0.6 (0.89)	2	0
Aesthetic and minimalist design	Not all field labels are brief, familiar, or descriptive Large objects, bold fonts, and simple areas have not been used to distinguish sections There is not enough white space between color representation Too much text is present in the keys which makes the screens look busy	0.8 (0.84)	2	0 and 1
Spatial organization	Font size is too small throughout the dashboard Information does not follow a logical flow	0 (0.0)	0	0
Information coding	No problems identified	0 (0.0)	0	0
Orientation	Measurement units are not displayed clearly Users cannot control the level of detail they see in a representation	0.6 (1.3)	3	0

The experts provided several recommendations for how to remedy the problems identified during the heuristic evaluation. They believed adding hyperlink functions would improve users’ ability to navigate between the dashboard views, thus increasing usability scores for visibility of system status, user control and freedom, and flexibility and efficiency of use. Aesthetically, the experts believed increasing the font size of titles and data within the dashboard, as well as moving different components of the dashboard around to maximize white space would improve scores for spatial organization, aesthetic and minimalist design, recognition rather than recall, and visibility of system status.

HCI SMEs also made recommendations that disagreed with user preferences identified during the design sessions. These differences included the experts preferring horizontal bar charts compared to vertical bar charts. An expert explained the rationale for this preference stating a horizontal bar chart allows for the labels in the table to be read horizontally, which can be easier for users. However, during the user-centered design sessions, the participants had a resounding preference for the vertical bar charts because they were more familiar with that format since some of their preexisting dashboards included vertical bar charts in the IPLS. Given the importance of the new dashboard to mimic the look of the existing ones, as well as the fact that the users explicitly preferred the vertical presentation, KD chose to retain the vertical bar charts. The color of the tables and graphs was also a point of disagreement between experts and users. The experts did not think the red, yellow, and green color choices were appropriate because these colors can be difficult to distinguish if individuals are color blind [39]. However, the targeted end users of the dashboard preferred those colors because the colors reminded them of traffic lights, and the users could make assumptions related to the significance of the colors based on what those colors mean by a traffic light. The research team chose to keep the traffic light color scheme.

**Figure 3:**
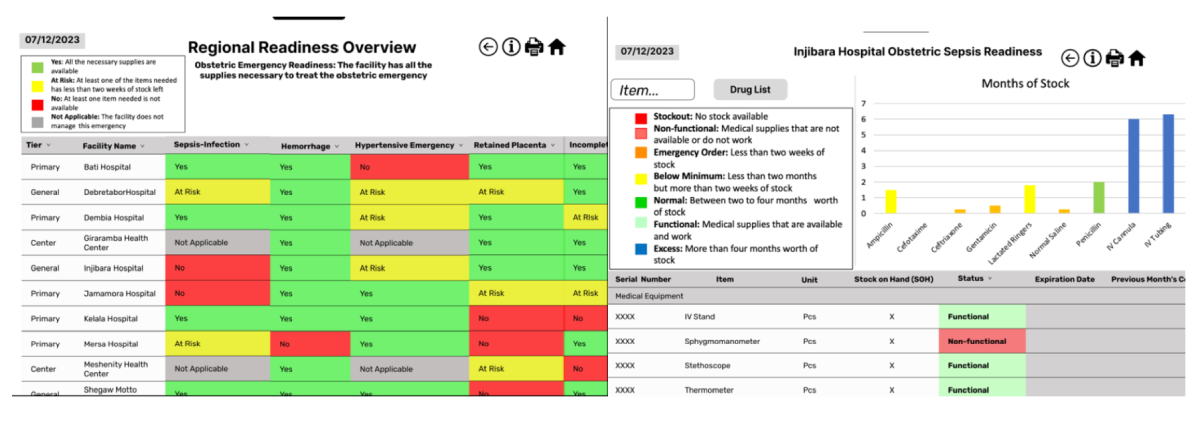
Final regional view and emergency-specific view of the dashboard following the heuristic usability evaluation. Hospital names are real, but all readiness data are fictitious.

Beyond these recommendations, the reviewers cited several positive features of the dashboard. This includes things such as the belief that the icons used throughout the dashboard were clear and easy to associate with their function. In addition, the experts found the labels and keys to be clear and assisted with comprehension of the data. One respondent reported, “Your labels are quite clear. It’s [the dashboard] very accessible and you have nice keys right here.” The heuristic violation severity scores for information visualization-specific heuristics were low with scores of zero for spatial organization and information coding, and 0.6 for orientation. Table 4 summarizes the changes incorporated into the dashboard following the completion of the heuristic usability evaluation. The final dashboard is displayed in Figure 3.

**Table 4:** Summary of changes incorporated into the dashboard following the heuristic evaluation

Heuristic category	Changes following heuristic evaluation
Visibility of system status	Increased font size throughout the dashboard Added hyperlinks to the dashboard
Match between system and real-world	Updated section headings to reflect filtering capabilities Updated the terminology defining BEmOCa readiness on the regional-level dashboard
User control and freedom	Added hyperlinks to the dashboard Ensured exit buttons on every screen are activated Activated more functions within the dashboard to make it a higher-fidelity prototype
Consistency and standards	Updated section headings to reflect filtering capabilities Defined all abbreviations used in the dashboard Changed the colors for functional and nonfunctional on the emergency-specific screen so that they are more easily distinguishable from the other colors used on the screen Added additional visual cues to identify active screens
Recognition rather than recall	Moved graphics at the top of the dashboard to optimize white space Added additional visual cues to identify active screens
Flexibility and efficiency of use	Added hyperlinks to the dashboard
Aesthetic and minimalist design	Shortened information in the keys to make them one line of text Shortened data within the tables to make it one line of text Used bold fonts for all titles Moved graphics at the top of the dashboard to optimize white space
Spatial organization	Increased font size throughout the dashboard Improved the navigation between dashboard views to make them a higher-fidelity prototype
Information coding	N/Ab
Orientation	Increased font size for measurement units Activated more functions within the dashboard to make it a higher-fidelity prototype

^a^BEmOC: basic emergency obstetric care.

^b^N/A: not applicable.

## Discussion

### Principal Findings

The dashboard underwent iterative refinement based on user input, domain expert feedback, information visualization evaluations, and heuristic usability assessments. Regional participants emphasized preferences for vertical bar charts, traffic-light color coding, and keys to define readiness levels, while facility-level users focused on aligning terminology with existing workflows and tailoring readiness classifications. The information domain expert provided critical input on readiness terminology, and information visualization experts suggested improvements in layout, navigation, and aesthetics, such as optimizing white space and increasing font sizes. Collectively, the central tendency of heuristic severity scores, combined with a mode of zero, indicated high overall usability of the dashboard. However, the HCI experts did highlight minor usability concerns, including navigation and accessibility improvements, which were addressed in the final design. While some expert recommendations, such as horizontal bar charts and alternative color schemes, differed from user preferences, the team prioritized end-user input to ensure practical usability. The final dashboard is intuitive, visually clear, and aligned with real-world needs, achieving high usability and user satisfaction.

This study highlights the importance of including participants from all targeted end-user groups. By including employees from both regional and facility levels, the team gathered diverse, relevant perspectives, ensuring the dashboard met the real-world needs of users across different supply chain roles and experiences. Feedback from both end users and experts enhanced the overall usability and functionality of the dashboard. This aligns with existing usability literature, which emphasizes the value of involving both end users and experts to identify distinct concerns and improve HIT design [40]. Participants’ spontaneous interpretations of the dashboard underscore their understanding of the content, and obtaining these perceptions and insights can assist in preventing usability errors from occurring later in the piloting or implementation phase of HIT development. These refinements will likely maximize the ease of implementation and promote consistent uptake and utilization by future users [41,42].

There were 2 areas of disagreement between HCI SMEs’ recommendations and end-user preferences: bar chart orientation and color schemes. The research team chose to keep the vertical bar charts for their familiarity with the end users and alignment with existing dashboards. In addition, lower color blindness prevalence among Black African and African American populations (4.2% compared to 6.6% and 1.4% compared to 5.6% respectively) compared to White populations (6.6% and 5.6%) [43,44] supported the decision to maintain the preferred color scheme, as most targeted end users were unlikely to face difficulties distinguishing the colors.

This study reinforces the notion that obtaining expert opinions and performing usability testing can be a low-cost, efficient method for exploring technology needs [45]. Performing the evaluations with the domain expert and the information visualization SMEs took less than an hour for both groups and provided critical information on how to improve upon the design of the dashboard. Performing usability evaluations can identify problems and usability concerns that may not have been noticed before HIT implementation, avoiding costly and time-consuming corrections [24].

Finally, this study contributes to the growing body of literature on the use of dashboards to support BEmOC in sub-Saharan Africa. Similar to findings by Banke-Thomas et al [46] and Wang et al [47], stakeholders expressed enthusiasm for dashboards and prioritized features like reporting facility characteristics on the dashboards. However, there remains a gap in the literature regarding the specific design preferences of end users. This study addresses that gap by highlighting key preferences, such as the use of stoplight color coding and IPLS-specific terminology, which enhance usability and are tailored specifically for dashboards intended for Ethiopia and BEmOC contexts.

### Limitations

During data collection, there was unanticipated civil unrest in the Amhara region, which prevented the research team from recruiting participants outside of the regional capital of Bahir Dar and from completing participant recruitment from multiple facilities outside the regional capital. Despite this unforeseen barrier, design saturation was reached for 2 out of the 3 categories during the design sessions. There was also a lack of gender diversity among our participants. Only one user-centered design participant from Ethiopia was female. However, this gender imbalance mirrored the existing gender imbalance in the existing health system for these roles. However, future research related to this dashboard and facility-level BEmOC readiness would benefit from an emphasis on opinions and participation from female participants with relevant expertise. Conversely, all HCI SMEs were female. While the incorporation of a female perspective is critical since the sample for user-centered design sessions was predominately male, the effect of having completely female HCI SMEs is unknown. Future research should incorporate technical and gender-balanced perspectives during both user and HCI SMEs’ review of this dashboard.

Furthermore, the dashboard design was informed by responses from participants in qualitative interviews. However, due to time constraints, the initial prototype was developed by the research team rather than being cocreated with participants. While this approach allowed the team to efficiently move forward with development, it may have limited the incorporation of deeper, iterative feedback from participants during the initial design phase. This limitation could have influenced the alignment of the prototype with user needs and preferences, which may require further refinement in future iterations.

To prospectively limit social desirability bias, the research team created a low-stress, comfortable environment for data collection and emphasized our desire to obtain personal responses to the dashboard while reiterating that all opinions would be blinded so no one could identify specific feedback from specific participants. The team chose to have an Amharic-speaking Ethiopian team member conduct the user-centered design sessions rather than the principal investigator, who is from the United States and does not speak Amharic. The team took steps to ensure confidentiality and used indirect questioning techniques in an attempt to garner the participants’ true opinions [48]. Furthermore, the dashboard reviewed in the heuristic usability evaluation was medium fidelity. This means the dashboard was not able to complete all required tasks and certain usability heuristics, such as *help and documentation* and *error prevention*, were excluded from this evaluation, since their tasks were not applicable at this stage in the design process. Future research will need to explore all usability heuristics before implementation to ensure no new concerns arise once the dashboard transitions from a medium- to high-fidelity model.

### Conclusion

In conclusion, this study demonstrates the value of user-centered design and usability evaluations in developing HIT for low-resource settings. By integrating diverse perspectives from regional and facility-level participants, as well as domain and usability experts, the dashboard was refined to meet real-world needs and achieve high usability. This iterative approach not only addressed usability concerns but also incorporated end-user preferences, ensuring alignment with existing workflows. The study fills a gap in the literature by identifying design preferences tailored to Ethiopia’s health care context, offering a model for future HIT development aimed at improving BEmOC readiness and maternal health outcomes. These findings emphasize the importance of engaging end users and experts early in the design process to create functional, user-friendly systems that support effective implementation and long-term adoption.

## Appendix

Multimedia Appendix 1 The graph options and preliminary dashboard prototypes used in the user-centered design sessions.

Multimedia Appendix 2 Heuristic usability evaluation survey.

Multimedia Appendix 3 Changes incorporated into the dashboard following the user-centered design sessions.

## Abbreviations

BEmOC: basic emergency obstetric care
HCI: human-computer interaction
HIPAA: Health and Insurance Portability Accountability Act
HIT: health information technology
IPLS: Integrated Pharmaceutical Logistics System
IRB: institutional review board
SME: subject matter expert

## References

[1] (2021). Trends in maternal mortality 2000 to 2017: estimates by WHO, UNICEF, UNFPA, World Bank Group and the United Nations Population Division. World Health Organization.

[2] Emory-Ethiopia partnership. Emory University's Nell Hodgson Woodruff School of Nursing.

[3] Tessema GA, Laurence CO, Melaku YA, Misganaw A, Woldie SA, Hiruye A, Amare AT, Lakew Y, Zeleke BM, Deribew A (2017). Trends and causes of maternal mortality in Ethiopia during 1990-2013: findings from the Global Burden of Diseases Study 2013. BMC Public Health.

[4] Dougherty K, Gebremariam Gobezayehu A, Lijalem M, Alamineh Endalamaw L, Biza H, Cranmer JN (2023). Comparison of obstetric emergency clinical readiness: a cross-sectional analysis of hospitals in Amhara, Ethiopia. PLoS One.

[5] Brenner SK, Kaushal R, Grinspan Z, Joyce C, Kim I, Allard RJ, Delgado D, Abramson EL (2016). Effects of health information technology on patient outcomes: a systematic review. J Am Med Inform Assoc.

[6] Ngwa W, Olver I, Schmeler KM (2020). The use of health-related technology to reduce the gap between developed and undeveloped regions around the globe. Am Soc Clin Oncol Educ Book.

[7] (2020). Fact sheet Ethiopia. USAID.

[8] Rabiei R, Almasi S (2022). Requirements and challenges of hospital dashboards: a systematic literature review. BMC Med Inform Decis Mak.

[9] Jbaily A, Feldhaus I, Bigelow B, Kamareddine L, Tolla MT, Bouvier M, Kiros M, Verguet S (2020). Toward health system strengthening in low- and middle-income countries: insights from mathematical modeling of drug supply chains. BMC Health Serv Res.

[10] Till S, Mkhize M, Farao J, Shandu LD, Muthelo L, Coleman TL, Mbombi M, Bopape M, Klingberg S, van Heerden A, Mothiba T, Densmore M, Verdezoto Dias NX (2023). Digital health technologies for maternal and child health in Africa and other low- and middle-income countries: cross-disciplinary scoping review with stakeholder consultation. J Med Internet Res.

[11] Bukachi F, Pakenham-Walsh N (2007). Information technology for health in developing countries. Chest.

[12] Lewis T, Synowiec C, Lagomarsino G, Schweitzer J (2012). E-health in low- and middle-income countries: findings from the center for health market innovations. Bull World Health Organ.

[13] Bekele TA, Gezie LD, Willems H, Metzger J, Abere B, Seyoum B, Abraham L, Wendrad N, Meressa S, Desta B, Bogale TN (2024). Barriers and facilitators of the electronic medical record adoption among healthcare providers in Addis Ababa, Ethiopia. Digit Health.

[14] Usability of consumer products and products for public use-part 2:summative test method. International Organization for Standardization.

[15] Abugabah A, Alfarraj A (2015). Issues to consider in designing health care information systems: a user-centred design approach. Electronic Journal of Health Informatics.

[16] Gnanlet A, Choi M, Davoudpour S (2019). Impediments to the implementation of healthcare information technology : a systematic literature review. Journal of Supply Chain and Operations Management.

[17] (2020). Standard operating procedures (SOP) manual for the Integrated Pharmaceuticals Logistics System in health facilities of Ethiopia. International Institute for Primary Health Care - Ethiopia.

[18] Dougherty K, Gebremariam A, Biza H, Belew M, Benda N, Tesfaye Y, Cranmer J, Bakken S (2024). Obstetric emergency supply chain dynamics and information flow among obstetric emergency supply chain employees: key informant interview study. JMIR Form Res.

[19] Dowding D, Merrill JA (2018). The development of heuristics for evaluation of dashboard visualizations. Appl Clin Inform.

[20] Nielsen J (1994). 10 Usability heuristics for user interface design. Nielsen Norman Group.

[21] Faulkner L (2003). Beyond the five-user assumption: benefits of increased sample sizes in usability testing. Behav Res Methods Instrum Comput.

[22] Guest G, Bunce A, Johnson L (2006). How many interviews are enough?. Field Methods.

[23] Allen M, Currie LM, Bakken S, Patel VL, Cimino JJ (2006). Heuristic evaluation of paper-based Web pages: a simplified inspection usability methodology. J Biomed Inform.

[24] Nielsen J (1992). Finding usability problems through heuristic evaluation. https://doi.org/10.1145/142750.142834.

[25] Darko EM, Kleib M, Olson J (2022). Social media use for research participant recruitment: integrative literature review. J Med Internet Res.

[26] Manji K, Hanefeld J, Vearey J, Walls H, de Gruchy T (2021). Using WhatsApp messenger for health systems research: a scoping review of available literature. Health Policy Plan.

[27] (2021). Medicine. Addis Ababa University.

[28] (2021). Nursing. Addis Ababa University.

[29] Lai TY, Larson EL, Rockoff ML, Bakken S (2008). User acceptance of HIV TIDES--tailored interventions for management of depressive symptoms in persons living with HIV/AIDS. J Am Med Inform Assoc.

[30] Lucero RJ, Sheehan B, Yen PY, Velez O, Nobile-Hernandez DL, Tiase VL, Bakken S (2012). Developing self-management tools with vulnerable populations for use in personal health information management systems. NI 2012 (2012).

[31] Vélez O, Okyere PB, Kanter AS, Bakken S (2014). A usability study of a mobile health application for rural Ghanaian midwives. J Midwifery Womens Health.

[32] Yen PY, Bakken S (2009). A comparison of usability evaluation methods: heuristic evaluation versus end-user think-aloud protocol - an example from a web-based communication tool for nurse scheduling. AMIA Annu Symp Proc.

[33] Arcia A, Benda N Visualization Design Studio.

[34] (2016). Figma.

[35] Nielsen J, Molich R (1990). Heuristic evaluation of user interfaces. https://doi.org/10.1145/97243.97281.

[36] Lai TY, Bakken S (2006). Heuristic evaluation of HIV-TIDES - tailored interventions for management of depressive symptoms in HIV-infected individuals. AMIA Annu Symp Proc.

[37] Choi J, Bakken S (2010). Web-based education for low-literate parents in neonatal intensive care unit: development of a website and heuristic evaluation and usability testing. Int J Med Inform.

[38] Alene GD, Ayenew GM, Bantie GM, Zeru T, Yismaw G (2023). Impact of ware on maternal health services in the war affected areas of the Amhara region, ethiopia: disruption of antenatla care, institutional delivery and postnatal services. Ethiopian Journal of Translational Sciences.

[39] Types of color vision deficiency. National Eye Institute.

[40] Følstad A (2017). Users’ design feedback in usability evaluation: a literature review. Hum Cent Comput Inf Sci.

[41] (2010). Ergonomics of human-system interaction part 210: human-centred design for interactive systems. International Organization for Standardization.

[42] Lau MK, Bounthavong M, Kay CL, Harvey MA, Christopher MLD (2019). Clinical dashboard development and use for academic detailing in the U.S. department of veterans affairs. J Am Pharm Assoc (2003).

[43] Mashige KP, van Staden DB (2019). Prevalence of congenital colour vision deficiency among Black school children in Durban, South Africa. BMC Res Notes.

[44] Color blindness most common in White boys. Review of Optometry.

[45] Farzandipour M, Nabovati E, Sadeqi Jabali M (2022). Comparison of usability evaluation methods for a health information system: heuristic evaluation versus cognitive walkthrough method. BMC Med Inform Decis Mak.

[46] Banke-Thomas A, Abejirinde IO, Ogunyemi O, Gwacham-Anisiobi U (2023). Innovative dashboard for optimising emergency obstetric care geographical accessibility in Nigeria: qualitative study with technocrats. Health Policy and Technology.

[47] Wang J, Wong KLM, Olubodun T, Gwacham-Anisiobi U, Ogunyemi O, Afolabi BB, Macharia PM, Makanga PT, Abejirinde IO, Beňová L, Banke-Thomas A (2023). Developing policy-ready digital dashboards of geospatial access to emergency obstetric care: a survey of policymakers and researchers in sub-Saharan Africa. Health Technol.

[48] Cobo B, Castillo E, López-Torrecillas F, Rueda MDM (2021). Indirect questioning methods for sensitive survey questions: modelling criminal behaviours among a prison population. PLoS One.

