# Supplementary material for: User-Centered Design of an Electronic Dashboard for Monitoring Facility-Level Basic Emergency Obstetric Care Readiness in Amhara, Ethiopia: Mixed Methods Study
Source: JMIR Hum Factors. 2025 Apr 3;12:e64131. doi: 10.2196/64131 (PMC12006772; doi:10.2196/64131)
Supplement: Multimedia Appendix 1 [file humanfactors_v12i1e64131_app1.docx]

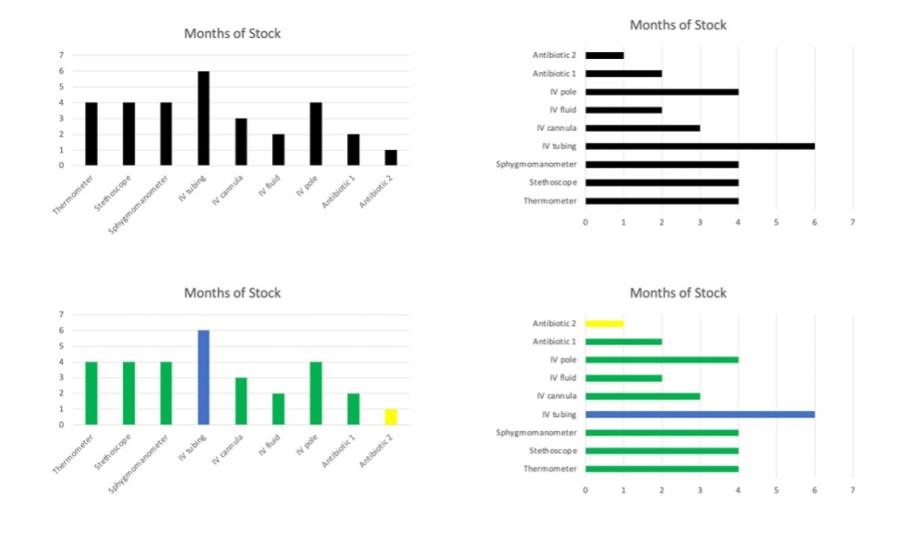


**Figure S1: Bar Chart Options to Represent Months of Stock Available**

*Note.* All inventory data in these graphics are fictitious.


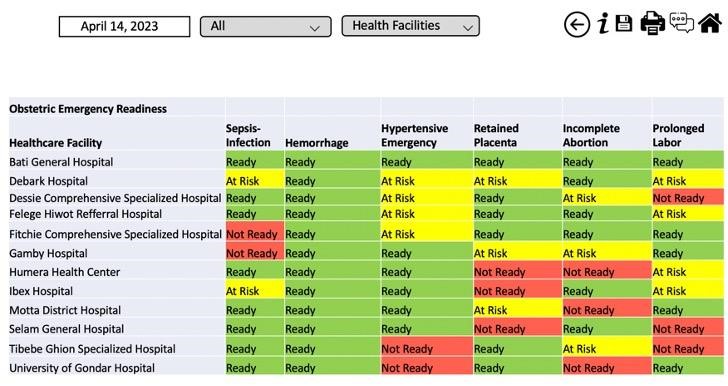


**Figure S2: Regional BEmOC Readiness Dashboard**

*Note.* Hospital names are real, but all readiness data is fictitious.


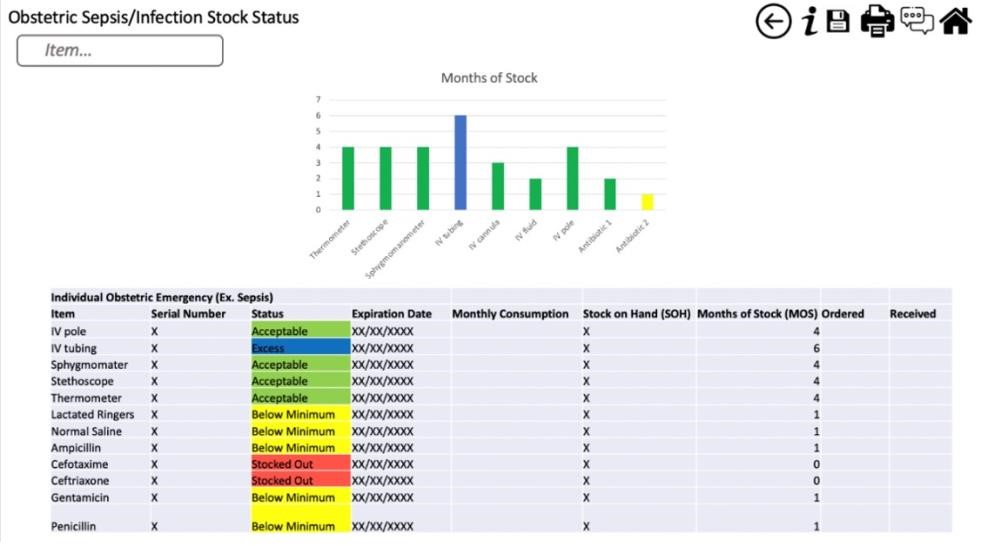


**Figure S3: Facility-Level Emergency-Specific Dashboard**

*Note.* All inventory data in this dashboard is fictitious.
