# Supplementary material for: User-Centered Design of an Electronic Dashboard for Monitoring Facility-Level Basic Emergency Obstetric Care Readiness in Amhara, Ethiopia: Mixed Methods Study
Source: JMIR Hum Factors. 2025 Apr 3;12:e64131. doi: 10.2196/64131 (PMC12006772; doi:10.2196/64131)
Supplement: Multimedia Appendix 2 [file humanfactors_v12i1e64131_app2.docx]

1. Visibility of System Status

The system should always keep user informed about what is going on, through appropriate feedback within reasonable time.

1. Please select your response for the individual items related to this usability factor:

| # | Usability Factor | Response | Comments |
| --- | --- | --- | --- |
| 1.1 | Does every screen have a title or header that describes its content? | - Yes - No - NA |  |
| 1.2 | Is there visual feedback in menus or dialog boxes about which choices are selectable? | - Yes - No - NA |  |
| 1.3 | Is there a clear indication of the current location? | - Yes - No - NA |  |
| 1.4 | Is the menu-naming terminology consistent with the user’s task domain? | - Yes - No - NA |  |
| 1.5 | Does the system provide *visibility*: that is, by looking, can the user tell the state of the system and the alternatives for action? | - Yes - No - NA |  |

1. Please select the overall severity rating for this usability factor:
2. If you have other comments, please specify.
3. Match between System and the Real-World

The system should speak the user’s language, with words, phrases and concepts familiar to the user, rather than system-oriented terms. Follow real-world conventions, making information appear in a natural and logical order.

1. Please select your response for the individual items related to this usability factor:

| # | Usability Factor | Response | Comments |
| --- | --- | --- | --- |
| 2.1 | Are the section headings and sub-sections in each screen ordered in the most logical way? | - Yes - No - NA |  |
| 2.2 | Is there a natural sequence to the menu choices for a data item? | - Yes - No - NA |  |
| 2.3 | Are all the words/concepts and phrases used in each screen familiar to users? | - Yes - No - NA |  |
| 2.4 | Are icons concrete and familiar? | - Yes - No - NA |  |
| 2.5 | Do the selected colors correspond to common expectations about color codes? | - Yes - No - NA |  |

1. Please select the overall severity rating for this usability factor:

| No usability Problem | Cosmetic Problem Only | Minor Usability Problem | Major Usability Problem | Usability Catastrophe |
| --- | --- | --- | --- | --- |
| 0 | 1 | 2 | 3 | 4 |

1. If you have other comments, please specify.
2. User Control and Freedom

Users should be free to select and sequence tasks (when appropriate), rather than having the system do this for them. Users will need a clearly marked “emergency exit” to leave the unwanted state without having to go through an extended dialogue. Users should make their ow decisions regarding the costs of exiting current work.

1. Please select your response for the individual items related to this usability factor:

| # | Usability Factor | Response | Comments |
| --- | --- | --- | --- |
| 3.1 | Is there a clear exit on each document screen? | - Yes - No - NA |  |
| 3.2 | Are all screens accessible across the system? | - Yes - No - NA |  |
| 3.3 | Can users easily move forward and backward between fields? | - Yes - No - NA |  |

1. Please select the overall severity rating for this usability factor:

| No usability Problem | Cosmetic Problem Only | Minor Usability Problem | Major Usability Problem | Usability Catastrophe |
| --- | --- | --- | --- | --- |
| 0 | 1 | 2 | 3 | 4 |

1. If you have other comments, please specify.

| No usability Problem | Cosmetic Problem Only | Minor Usability Problem | Major Usability Problem | Usability Catastrophe |
| --- | --- | --- | --- | --- |
| 0 | 1 | 2 | 3 | 4 |

1. Consistency and Standards

Users should not have to wonder whether different words, situations, or actions mean the same thing.

1. Please select your response for the individual items related to this usability factor:

| # | Usability Factor | Response | Comments |
| --- | --- | --- | --- |
| 4.1 | Have formatting standards been followed consistently in all screens within the system? | - Yes - No - NA |  |
| 4.2 | Are abbreviations clearly explained? | - Yes - No - NA |  |
| 4.3 | Are there salient visual cues to identify the active screen? | - Yes - No - NA |  |
| 4.4 | Is vertical scrolling possible in each screen? | - Yes - No - NA |  |
| 4.5 | Are there no more than four to seven colors, and are they far apart along the visible spectrum? | - Yes - No - NA |  |
| 4.6 | Is the most important information placed at the beginning of the form? | - Yes - No - NA |  |
| 4.7 | Are names consistent, both within each tab and across the system, in grammatical style and terminology? | - Yes - No - NA |  |
| 4.8 | Is there a consistent icon design scheme and stylistic treatment across the system? | - Yes - No - NA |  |

1. Please select the overall severity rating for this usability factor:

| No usability Problem | Cosmetic Problem Only | Minor Usability Problem | Major Usability Problem | Usability Catastrophe |
| --- | --- | --- | --- | --- |
| 0 | 1 | 2 | 3 | 4 |

1. If you have other comments, please specify.
2. Recognition Rather than Recall

Make objects, actions, and options visible. The user should not have to remember information from one part of the dialogue to another. Instructions for use of the system should be visible or easily retrievable whenever appropriate.

1. Please select your response for the individual items related to this usability factor:

| # | Usability Factor | Response | Comments |
| --- | --- | --- | --- |
| 5.1 | Are prompts, cues, and messages placed where the eye is likely to be looking on the screen? | - Yes - No - NA |  |
| 5.2 | Is white space used to create symmetry and lead the eye in the appropriate direction? | - Yes - No - NA |  |
| 5.3 | Have items been grouped into logical zones, and have headings been used to distinguish between zones? | - Yes - No - NA |  |
| 5.4 | Is color highlighting used to get the user’s attention? | - Yes - No - NA |  |
| 5.5 | Is color coding consistent throughout the system? | - Yes - No - NA |  |
| 5.6 | Can the user easily locate data? | - Yes - No - NA |  |

1. Please select the overall severity rating for this usability factor:

| No usability Problem | Cosmetic Problem Only | Minor Usability Problem | Major Usability Problem | Usability Catastrophe |
| --- | --- | --- | --- | --- |
| 0 | 1 | 2 | 3 | 4 |

1. If you have other comments, please specify.
2. Flexibility and Efficiency of Use

The website should offer users a number of options when it comes to finding content on the site. Users should be able to achieve their goals in an efficient manner.

| # | Usability Factor | Response | Comments |
| --- | --- | --- | --- |
| 6.1 | Is navigation between screens simple and visible? | - Yes - No - NA |  |

1. Please select the overall severity rating for this usability factor:

| No usability Problem | Cosmetic Problem Only | Minor Usability Problem | Major Usability Problem | Usability Catastrophe |
| --- | --- | --- | --- | --- |
| 0 | 1 | 2 | 3 | 4 |

1. If you have other comments, please specify.
2. Aesthetic and Minimalist Design

Dialogues should not contain information which is irrelevant or rarely needed. Every extra unit of information in a dialogue competes with the relevant units of information and diminishes their relative visibility.

1. Please select your response for the individual items related to this usability factor:

| # | Usability Factor | Response | Comments |
| --- | --- | --- | --- |
| 7.1 | Is only (and all) information essential to decision making displayed on the screen? | - Yes - No - NA |  |
| 7.2 | Have large objects, bold fonts, and simple areas been used to distinguish sections? | - Yes - No - NA |  |
| 7.3 | Are field labels brief, familiar, and descriptive? | - Yes - No - NA |  |
| 7.4 | Is the visual layout well designed? | - Yes - No - NA |  |
| 7.5 | Are there any unnecessary data elements in each screen? | - Yes - No - NA |  |
| 7.6 | Is each lower-level sub-section/data item associated with only one higher-level section? | - Yes - No - NA |  |
| 7.7 | Is data presented in a simple format? | - Yes - No - NA |  |
| 7.8 | Is there white space between color representation? | - Yes - No - NA |  |

1. Please select the overall severity rating for this usability factor:

| No usability Problem | Cosmetic Problem Only | Minor Usability Problem | Major Usability Problem | Usability Catastrophe |
| --- | --- | --- | --- | --- |
| 0 | 1 | 2 | 3 | 4 |

1. If you have other comments, please specify.
2. Spatial Organization

Relates to the overall layout of a visual representation and refers to how easily it is to locate an information element in the display and the distribution of elements in the representations.

1. Please select your response for the individual items related to this usability factor:

| # | Usability Factor | Response | Comments |
| --- | --- | --- | --- |
| 8.1 | Are all information elements clear and visible? | - Yes - No - NA |  |
| 8.2 | Does the information follow a “logical” organization? | - Yes - No - NA |  |
| 8.3 | Does the information provide detail on the context and detail associated with the data element? | - Yes - No - NA |  |

1. Please select the overall severity rating for this usability factor:

| No usability Problem | Cosmetic Problem Only | Minor Usability Problem | Major Usability Problem | Usability Catastrophe |
| --- | --- | --- | --- | --- |
| 0 | 1 | 2 | 3 | 4 |

1. If you have other comments, please specify.
2. Information Coding

Relates to the symbols or representations used to aid perception.

1. Please select your response for the individual items related to this usability factor:

| # | Usability Factor | Response | Comments |
| --- | --- | --- | --- |
| 9.1 | Are the symbols appropriate for the data represented? | - Yes - No - NA |  |
| 9.2 | Are realistic characteristics used to represent data or information elements? | - Yes - No - NA |  |

1. Please select the overall severity rating for this usability factor:

| No usability Problem | Cosmetic Problem Only | Minor Usability Problem | Major Usability Problem | Usability Catastrophe |
| --- | --- | --- | --- | --- |
| 0 | 1 | 2 | 3 | 4 |

1. If you have other comments, please specify.
2. Orientation

Provision of support for the user to orient them in the visualization.

1. Please select your response for the individual items related to this usability factor:

| # | Usability Factor | Response | Comments |
| --- | --- | --- | --- |
| 10.1 | Are the measurement units displayed clearly? | - Yes - No - NA |  |
| 10.2 | Are there labels associated with each data field? | - Yes - No - NA |  |
| 10.3 | Can the user control the level of detail they see in a representation? | - Yes - No - NA |  |

1. Please select the overall severity rating for this usability factor:

| No usability Problem | Cosmetic Problem Only | Minor Usability Problem | Major Usability Problem | Usability Catastrophe |
| --- | --- | --- | --- | --- |
| 0 | 1 | 2 | 3 | 4 |

1. If you have other comments, please specify.
