# Supplementary material for: User-Centered Design of an Electronic Dashboard for Monitoring Facility-Level Basic Emergency Obstetric Care Readiness in Amhara, Ethiopia: Mixed Methods Study
Source: JMIR Hum Factors. 2025 Apr 3;12:e64131. doi: 10.2196/64131 (PMC12006772; doi:10.2196/64131)
Supplement: Multimedia Appendix 3 [file humanfactors_v12i1e64131_app3.docx]

**Changes incorporated into the dashboard following the user-centered design session**

**Aesthetic changes incorporated into the dashboard following user-centered design sessions**

| **Change Recommended** | **Version** | **Session** | **Level** | **Quote** |
| --- | --- | --- | --- | --- |
| Changed to vertical, color-coded bar charts | 1 | 1, 2 | Regional | *“There could be one color for excess products, one color for maximum stock until minimum stock, one color for minimum stock level, which should have its own color, one color for below minimum stock, which is an emergency, and another color for stockout.” -Regional*  *“The usual one is the vertical bars” -Regional* |
| Moved serial number column to be first | 1 | 1 | Regional | *“First serial number, then the product name” -Regional* |
| Added a key for readiness levels | 1 | 1, 2 | Regional | *“A key [needs to] be presented here that explains the meanings of ‘ready,’ ‘at risk’” -Regional*  *“The written word and color both contribute to the description.” -Regional* |
| Added a key for quantity status | 1 | 1, 2 | Regional | *“Key at the top would help.” -Regional*  *“The need for operational definitions is something else we are stating. It will be straightforward if there is an operational definition given for these terms.” -Regional*  *“When we say ‘maximum’ in our case, for instance, we mean holding onto the stock for a minimum of two months and a maximum of four months. Therefore, it’s crucial to operationalize the terms so that you may… specific the minimum and maximum levels.” -Regional* |
| Added a button to view comprehensive drug list in the facility-view dashboard | 1 | 1, 2 | Regional | *“I don’t think we need that there; I believe there’s no need for a batch number or expiration date to be present here.” -Regional*  *“You can get it from Vitas, we frequently utilize this tool. Everything that is here is also there.” -Regional* |
| Removed the save button | 1 | 1, 2 | Regional | Designer decision following discussion with users about the tasks they would accomplish with the dashboard and data entry was not an included task |
| Removed request and received columns in the facility-view dashboard | 1 | 1, 2 | Regional | *“If we must use these columns then received must come around here (shows on image).” -Regional*  *“You can get it from Vitas, we frequently utilize this tool. Everything that is here is also there.” -Regional* |
| Greyed out the readiness classifications at the hub level for the emergencies they do not treat | 2 | 3 | Facility | *“There are services that are not provided by health posts. Since some of these services are not provided by health posts, in addition to the colors we use for readiness risk, we need to have a color that may show services not provided by health posts or we need to have a system that may hide it.”-Facility* |
| Increased font size for emergency-specific dashboard | 2 | 4 | Facility | *“It is difficult to read.” -Facility* |
| Removed medical supplies from bar chart in the facility-view dashboard | 2 | 3 | Facility | *“This is a good one [bar charts]. However, it only works for supplies; it cannot be utilized for medical equipment.” -Hospital* |
| Removed expiration date and months of stock data from medical supplies in the facility-view dashboard | 2 | 3 | Facility | *Respondent 1: “It appears here a month’s worth of stock, the status, expiration date, stock on hand, and prior month’s consumption, and average month’s consumption are all displayed…it is very good”*  *Respondent 2: “This is an excellent one; however, it doesn’t apply to medical equipment.” -Hospital* |
| Added additional medication strengths to the data table for the emergency-specific dashboard | 2 | 3 | Facility | *“Ampicillin is correct, but the strength should be written, like is it 500 mg, 300 mg, 250 mg, an injection?” -Facility* |

**Filtering and sorting changes incorporated into the dashboard following user-centered design sessions**

| **Change** | **Version** | **Session** | **Respondent Level** | **Quote** |
| --- | --- | --- | --- | --- |
| Selected the option to sort supplies by categories and alphabetically | 1 | 1, 2 | Regional | *“This can be useful for inventory purposes…they can see alphabetically to make counts.” -Regional*  *“It is beneficial and helpful to sort by category with level. The alphabetical order thus allows us to easily organize items. Consequently, it is beneficial if we employ both categories.” -Regional* |
| Provided a filtering option for supply quantities | 1 | 1, 2 | Regional | *“It is good if the dashboard also displays products at risk as well as overstock and understock.” -Regional*  *“I think the sorting option is better. If the products which products are overstocked so we can observe which products are in stock, then which products are at the emergency point.” -Regional*  *“We also need to prevent supply shortages; thus, it would be beneficial if we could immediately notice the at-risk item and they need to come first.”-Regional* |
| Provided a filtering option based on facility tier and make facility tier a new column | 1 | 1, 2 | Regional | *“Why don’t we add a column for primary, general, and other health facility rankings so that we may conveniently filter and export for later use?” -Regional*  *“This one [health facility drop-down] would be useful for finding a summary report, such as a summary report for a health post or a health center.” -Regional* |
| Allowed facilities to be able to view inventory data from other healthcare facilities in the region instead of only seeing the data from their facility | 2 | 4 | Facility | *“It is important for us to see other hospitals status like general and referral hospitals like [hospital] and…hospitals because they provide comprehensive care and give services like we do, and we can support one another. If you run out of supplies that are difficult to obtain, like dialysis catheters, you can get them from these facilities, which is crucial.” -Facility*  *“Keep in mind that the relationship between the health post and the health center is quite strong because the health post receives supplies from the health centers. It is acceptable to suggest that the health post and health center should appear in the dashboard as data or information.” -Facility* |

**Match with the real-world changes incorporated into the dashboard following user-centered design sessions**

| **Change** | **Version** | **Session** | **Respondent Level** | **Quote** |
| --- | --- | --- | --- | --- |
| Updated supply category terminology | 1 | 1, 2 | Regional | *“There are four categories at the Ministry of Health, and these are standard labels, and it is good if we use the standard labels that we have. Among the four, the first is ‘pharmaceuticals’, and medicine may be including IV fluids…the second group includes ‘medical supplies’…the third one that we can use to label is ‘reagents and chemicals’…then the fourth label, ‘medical equipment.’” -Regional*  *“These categories should exist. It will enable us to quickly pinpoint the issue. However, it is preferable if we stick to standard terminology.” -Regional* |
| Added *emergency order point* category to supply quantities | 1 | 2 | Regional | *“Maximum, minimum, and emergency order are used to categorize status in our organization.” -Regional* |
| Updated supply quantity categories | 1 | 2 | Regional | *“The words we routinely use on a national level, such as ‘okay,’ ‘overstock,’ ‘below minimum stock’ and so forth.” -Regional*  *“I believe it is preferable if we use similar terms, such as stockout, as opposed to stocked out.” -Regional* |
| Provided a definition for obstetric emergency | 1 | 2 | Regional | *“The phrase ‘obstetric emergency readiness’…is unclear to me…I believe that it needs to be adequately described.” -Regional* |
| Updated the definition of *below minimum* | 2 | 2, 3 | Facility | Designer decision to make sure there is no confusion in the definitions for *below minimum* and *emergency order* |
| Changed *IV pole* to *IV stand* | 2 | 3 | Facility | *“We must use conventional or standard terminology; for instance, you don’t use the term ‘iv pole’…it should say ‘iv stand.’”-Hospital* |
| Changed quantity status terminology for medical supplies to *functional* or *nonfunctional* and updated the key to reflect this change | 2 | 3 | Facility | *“The only thing is how can we say medical equipment is excess, at risk, this naming is not a problem for pharmaceuticals, but such naming may not work for equipment. It might be challenging to assess whether medical equipment is excessive or not, but the best way to talk about them is to determine first whether the medical equipment is functional or non-functional.” Hospital*  *“Instead of saying excess or minimum, we might state functional and nonfunctional for medical equipment when taking inventory.” Hospital* |
| Provided different terminology options for regional readiness | 2 | 3 | Facility | “*In our case, we say ‘normal’ for what you described as the green ‘ready’. Next, the others are stockout and below the emergency order point.” -Hospital* |
